# Positive Psychological Coaching Definitions and Models: A Systematic Literature Review

**DOI:** 10.3389/fpsyg.2020.00793

**Published:** 2020-05-06

**Authors:** Llewellyn E. van Zyl, Lara C. Roll, Marius W. Stander, Stefanie Richter

**Affiliations:** ^1^Department of Industrial Engineering, University of Eindhoven, Eindhoven, Netherlands; ^2^Optentia Research Focus Area, North-West University (VTC), Vanderbijlpark, South Africa; ^3^Department of Human Resource Management, University of Twente, Enschede, Netherlands; ^4^Institut für Psychologie, Goethe University, Frankfurt, Germany; ^5^Department of Applied Psychology, Lingnan University, Tuen Mun, Hong Kong; ^6^Faculty of Psychology, Technische Universität Dresden, Dresden, Germany

**Keywords:** positive psychological coaching, coaching psychology, strengths-based coaching, positive psychological interventions, coaching model, positive organizational interventions, performance enhancement

## Abstract

Despite the popularity of the term Positive Psychological Coaching within the literature, there is no consensus as to how it should be defined (framed) or what the components of a positive coaching “model” should include. The aim of this systematic review was to define positive psychological coaching and to construct a clear demarcated positive psychological coaching model based on the literature. A systematic literature review led to the extraction of 2,252 records. All records were screened using specific inclusion/exclusion criteria, which resulted in the exclusion of records based on duplicates (*n* = 1,232), titles (*n* = 895), abstracts (*n* = 78), and criteria violations (*n* = 23). Twenty-four academic, peer-reviewed publications on positive psychological coaching were included. Data relating to conceptual definitions and coaching models/phases/frameworks were extracted and processed through thematic content analysis. Our results indicate that positive psychological coaching can be defined as a short to medium term professional, collaborative relationship between a client and coach, aimed at the identification, utilization, optimization, and development of personal strengths and resources in order to enhance positive states, traits and behaviors. Utilizing Socratic goal setting and positive psychological evidence-based approaches to facilitate personal growth, optimal functioning, enhanced wellbeing, and the actualization of people's potential. Further, eight critical components of a positive psychological coaching model were identified and discussed. The definition and coaching process identified in this study will provide coaches with a fundamental positive psychological framework for optimizing people's potential.

## Background

Positive Psychological Coaching (also referred to as Strengths-Based Coaching, or Positive Coaching) has been positioned as a solution-focused “applied positive psychological approach” aimed at facilitating goal achievement, wellbeing and positive change in various life domains (Madden et al., 2011) and application areas (Castiello D'Antonio, 2018). This “positive” coaching approach is fuelled by recent developments in the strengths-literature, whereby an individual's signature strengths are used to facilitate the personal growth and development of a client^1^ (van Zyl and Stander, 2013). From this perspective, each individual's capacity for personal growth and goal achievement is a function of the identification, optimization and application of individual strengths (Linley and Joseph, 2004; Castiello D'Antonio, 2018). Focusing on the “positive” rather than fixing the “deficits” resonates with practitioners, as the focus is on development rather than deficit correction (Stander, 2016). Positive psychological coaching employs a comprehensive approach toward development, which aids clients to identify and actively deploy their character strengths as well as acknowledges the multiple contexts which influences their lives (Haberlin, 2019). Research showed that this novel approach to people development leads to various positive consequences for both the individual (e.g., improved performance, self-efficacy, life satisfaction, self-confidence etc.) and the organization (e.g., talent retention, employee engagement, customer satisfaction, financial growth etc.; Peláez et al., 2019). It is therefore not surprising that this positively framed approach has been popularized within practice and popular psychological press as an effective organizational or talent development intervention (Stander, 2016; Haberlin, 2019).

Despite the popularity of “strengths-based”- or “positive” coaching within the literature, there is still little consensus as to how it should be defined (framed) and what the components of a “positive coaching process” should involve (Peláez et al., 2019). Various *definitions* or conceptualizations of positive coaching approaches exist within the literature which differ significantly from one another. For example, Linley and Joseph (2004), (p. 4) argued that positive coaching is a process aimed at the promotion of optimal functioning across a full range of human capability. Where van Zyl and Stander (2013) defined it as a professional, client-centered relationship aimed at the identification, utilization, and optimization of individual strengths in order to facilitate the development of individuals and organizations. This definition is partially echoed by Grant et al. (2010) who postulated that positive coaching is a collaborative, solutions-orientated approach designed to facilitate the achievement of personal goals through capitalizing on individual strengths.

In contrast, Denison and Avner (2011) said that positive coaching concerns a set of behavioral guidelines which are easy to use, mechanistic and are formatted as best practice guidelines for what to do and what not to do within a given context. Orem et al. (2007) on the other hand argued that positive or “appreciative” coaching relates to the development of self-compassion through an appreciative relationship with a coach. Castiello D'Antonio (2018) further argued that positive psychological coaching is an approach which seeks to enhance the short-term hedonic- (life satisfaction) and sustainable long-term wellbeing (flourishing) of a client by using evidence-based positive psychological approaches. Although there is some overlap in how it is defined, significant differences in the conceptualization and approach toward positive psychological coaching exist within the literature. Moreover, these definitions are used by each of the respected authors as if agreement exists on the conceptualization of the construct, however these agreements are more often only shared between a specific set of authors rather than a matter of consensus. As such, the lack of a coherent theoretical conceptualization of the concept results in not only criticism of the paradigm, but also results in an inability to discern what constitutes positive coaching and what not (Wong and Roy, 2017).

The lack in consensus is not just confined to how positive psychological coaching is defined, but also as to how it is approached within practice. Specifically, there seems to be little consensus as to what constitutes a *positive psychological coaching model, -framework or -approach*. Anstiss and Passmore (2017) utilized the PERMA model of Seligman (2012) as a coaching framework indicating that coaches should aim to enhance positive emotional experiences, engagement, positive relationships, meaning and accomplishments of their clients. Freire (2013) on the other hand proposed that the “Authentic Happiness” framework of Seligman (2004) could act as a coaching model where clients are facilitated to enhance pleasure in the present/past/future, to increase engagement and to aid in finding meaning in life. However, Dyess et al. (2017) argued that these are merely outcomes of a coaching process and does not constitute a theoretically grounded coaching model or framework. Dyess et al. (2017) suggested that strengths-based coaching take the form of a 4-phased model starting off with (a) building trust, (b) naming the strengths of clients, (c) aiding clients to claim their strengths, and (d) aiming it at the right goals. This approach, however, fails to take the client's ideal or “best possible self” into consideration, which leads some authors to employ Appreciative Inquiry as an alternative coaching framework (Gordon and Gucciardi, 2011; Gordon, 2016). From this perspective, the focus is on identifying the best of what currently is or has been (Discovery), creating a clear vision of their ideal-selves (Dream), assisting the client to create possibilities to actualize their vision/goals (Design), and aiding clients to implement and track initiatives in order to achieve their dreams (Destiny) (Gordon and Gucciardi, 2011; Gordon, 2016).

Appreciative inquiry, however, only focuses on what currently works well, and negates opportunities to focus on the enhancement of developmental areas. As a result, Kauffman et al. (2015) as well as van Zyl and Stander (2013) argued for the adoption of solutions orientated approaches toward positive coaching. Both teams of authors argued for the identification of strengths/skills that could be used to not only achieve goals, but also to aid in closing the gaps between developmental areas and desired outcomes. van Zyl and Stander (2013) however presented a structured 10 phase model on how this could be achieved. They argued that clients need to be developed within the system in which they function, and therefore the organizational reality plays a major role in defining the coaching trajectory. In this strengths-based coaching model, the coach clarifies expectations with the client and his/her stakeholders, aids in the identification of signature strengths and developmental needs, which acts as the basis for deriving coaching themes (van Zyl et al., 2016b). Coaches aid in developing solutions-orientated goals, which stem from an ideal vision crafted by a client. Strengths and competencies are developed, and clients are empowered to reframe challenges/problems from a strengths-based perspective (Stander, 2016). Although these are just some examples of positive psychological coaching approaches/models, there are a magnitude of other approaches within the literature; yet no clear conceptual coaching framework exists.

The lack of both a clearly articulated definition and a process-orientated positive psychological coaching methodology/framework/model, leads some to believe that such is a product of pseudo-science, or that it is another victim of the jangle fallacy (i.e., the assumption that it may be an old concept in a proverbially new jacket; Wong and Roy, 2017; Compton and Hoffman, 2019; Yakushko, 2019). For positive psychological coaching to distinguish itself from other approaches to coaching and to develop its own identity within science, there need to be an objective, systematically developed and organized body of knowledge supporting such. This knowledge should be available for other researchers to utilize, implement, validate, evaluate, critique, and update in an objective and systematic manner. The bases of such a body of knowledge starts with a widely accepted and standardized definition of the concept (Creswell, 2013). The lack of a standardized definition and approach may also negatively affect the effectiveness of positive psychological coaching interventions as these are therefore not built on validated empirical models or evidence-based theoretical frameworks (Compton and Hoffman, 2019). This in turn may be harmful to the client as failures in the coaching process may lead to “confirmation” of his/her subjectively perceived personal deficiencies (Wong and Roy, 2017).

As such, the purpose of this systematic review is to clarify the theoretical conceptualization of positive coaching, as well as its underlying components. Specifically, the aim is to construct a commonly shared understanding or “definition” of the concept, and to construct a clear demarcated positive psychological coaching model/process to aid in the development of people.

## Methods

### Research Approach

A systematic literature review was employed to determine how positive psychology coaching should be conceptualized, with the specific aim of constructing a definition, a coaching model and clarifying the components of such a coaching approach. A systematic literature review employs a systematic approach to identify, select and critically evaluate the available research on a given topic with the aim of synthesizing an answer to a clearly defined research question (Booth et al., 2016). For this systematic review, the “Preferred Reporting Items for Systematic Reviews and Meta-Analyses (PRISMA)” reporting guidelines were employed (Moher et al., 2009). The PRISMA guidelines provide a universally accepted evidence-based checklist of the components which need to be reported within a systematic literature review in order to enhance transparency, clarity and credibility. Derived from these guidelines, we developed and systematically applied a clear extraction and classification taxonomy aligned to the purpose of the study (Booth et al., 2016). This taxonomy dictated the extent of the search, the inclusion/exclusion criteria, how data is to be coded/analyzed and how disagreement between researchers should be managed (Booth et al., 2016).

### Search Strategy

**Figure 1:**
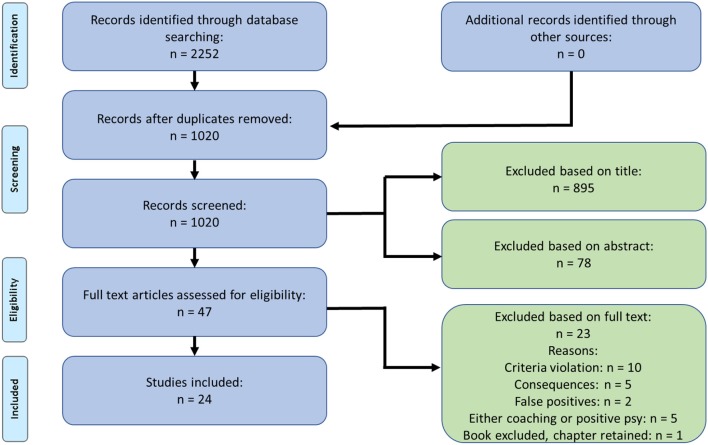
Flow diagram of article selection.

We conducted a comprehensive systematic literature search between April and July 2019 in the bibliographic databases Scopus, Web of Science, ScienceDirect, PsycInfo, and ACM Digital Library. In those databases we used nine primary search terms: “positive psychology coaching,” “strengths coaching,” “strengths-based coaching,” “positive coaching,” “positive therapy at work,” “positive personal development,” “integrative positive coaching,” “wellbeing coaching,” and “happiness coaching.” First, the primary search terms were applied, and second, in a subsequent search, the databases were queried with a combination of each primary term with the secondary terms “model OR process OR theory OR program” (e.g., “positive psychology coaching” AND “model OR process OR theory OR program”). Using these search terms, 2,252 titles were identified from 2000^2^ up until June 2019 (c.f. Figure 1 for the flow diagram of article selection).

### Eligibility Criteria

For manuscripts to be eligible for inclusion into the paper, a number of inclusion and exclusion criteria was set before the start of the project. *Included* manuscripts needed to (a) be academic peer-reviewed, theoretical articles with a clear focus on model- or theory construction, (b) the focus of these publications needed to be centered around positive coaching psychology, (c) these papers needed to be specifically aligned with the theoretical tradition of positive psychology and could emanate from any application field (e.g., sports or business), (d) only academic peer-reviewed scientific papers, books and book chapters published in English were eligible for inclusion, (e) manuscripts needed to be published in journals that were ISI, Web of Science and Scopus listed, and (f) the year of publication had to fall between 2000 and June 2019.

In contrast, we *excluded*: (1) Publications in non-English formats; (2) non-peer-reviewed books and articles (such as popular psychology- or management books and practitioner focused non-academic journals); (3) articles focusing on instrument development, empirical work or validations of a coaching intervention; (4) unpublished master and doctoral theses; (5) textbooks and conference proceedings; (6) any publications with a focus on non-psychological and/or non-behavioral coaching (such as physical strengths conditioning in body building); and (7) articles which focused on the outcomes of positive coaching rather than the process itself.

### Study Selection

After completing the search, the study selection process involved four distinct phases and was managed by all four authors. First, the titles of all the studies were tested against the eligibility criteria and screened for inclusion by the co-ordinating author and the three co-authors. Second, the relevant abstracts of all papers included via the title screening process were then extracted and screened for inclusion by the authors. Third, the full papers were then extracted and screened for final inclusion. Lastly, the final list of included papers was collated and circulated to five prominent academics within the field of positive psychology and positive coaching psychology to identify additional records; no additional records were added.

Our search revealed 2,252 records. After removing duplicates, our systematic literature search yielded 1,020 unique titles, from which a total of 24 publications were included in the final selection (Figure 1). We excluded 895 publications based on titles, 78 publications after reading their abstracts, and 23 publications based on their full texts. Criteria violation, e.g., empirical or textbook papers, was the most common reason for publications to be excluded (*n* = 10), followed by 5 publications excluded because of their focus being on consequences of positive psychology coaching. A total of 5 papers had to be excluded because their focus was either on coaching or on positive psychology, but the two perspectives were not combined. In two cases the abstracts contained our key words, yet they were not discussed in the main text. These papers were classified as false positives and removed. One book was excluded with only the one relevant chapter thereof retained. Following this screening of titles, abstracts and full texts, we continued with the final selection of 24 articles for further analysis. A full overview of the included papers and their purpose can be seen in Appendix A.

### Selection Bias

To manage selection bias and to enhance the credibility, conformability, and transparency of the systematic review, a number of strategies were employed. First, after the initial search by the co-ordinating author, one of the co-authors performed the literature search following the steps described in the study selection section above. This was done in order to ensure that no records were missed/excluded during the selection process (Moher et al., 2009). Secondly, each of the titles, abstracts and final papers were independently coded and scored by the co-ordinating author and one of the co-authors. At the completion of each phase, the co-authors would meet to debate the inclusion/exclusion of titles/abstracts/final papers. During these meetings, the reasons for exclusion of a record was discussed and noted. Here, Cohen's kappa coefficient was employed as a means to estimate the inter-rater reliability (McHugh, 2012). The following equation was used:

$$\begin{array}{l} k=\frac{\mathrm{Pr}\left(a\right)-\mathrm{Pr}\left(e\right)}{1-\mathrm{Pr}\left(e\right)} \end{array}$$

Cohen's kappa (κ) is a function of the relative observed agreement between the raters (Pr_α_) minus the hypothetical probability of agreement by chance (Pr_*e*_), divided by the standardized probability of chance (McHugh, 2012). Landis and Koch (1977) argued that a minimum kappa level of 0.61 would be considered acceptable. In order to calculate kappa, the Crosstabs function in SPSS was used. The results showed that that there was substantial agreement between raters (*k* = 0.87; *p* < 0.01) which exceeded the suggested values of Landis and Koch (1977).

Third, to increase the trustworthiness of the thematic content analyses and the coding process, the data was independently analyzed by the co-ordinating author and one of the authors for each of the two separate sections (definition and model). Here open communication between the co-ordinating author and the three co-authors persisted throughout the analysis process (Coetzee and van Zyl, 2014). Disagreements in the coding process were discussed between the two coders until it was resolved. In the few cases where agreement could not have been found, a third member of the team's opinion was sought. After the initial coding process was completed, a meeting was held whereby all authors had the chance to work through the codes and to discuss agreements/disagreements. The average level of agreement between coders exceeded Miles and Huberman's (1994) suggested 70%. All the raw and process data was retained for possible future scrutiny.

### Data Recording and Analyses

Data from the selected studies were extracted and captured verbatim onto a Microsoft Excel Spreadsheet for further processing. Most authors included information relating to the definition and models of positive psychological coaching in their introduction and discussion sections, though each whole article/chapter was screened for all information related to both topics. Marginal cases were discussed among the authors of this article until consensus was reached whether to include the information for further analysis.

Subsequently, data was processed through thematic content analysis (Creswell, 2013). This procedure allows the quantification of large quantities of textual information. Important text properties were systematically identified through structured categorization according to its relevancy to definition, coaching model, and coaching tools (Creswell, 2013; van Zyl, 2013). The advantages of this procedure include that it is non-intrusive (Duriau et al., 2007), highly flexible (Creswell, 2013), and results can be replicated and quantified in terms of frequencies/percentages (van Zyl, 2013). However, this type of analysis is subjected to the same limitations as traditional nominal-oriented measurement techniques (Salkind, 2012).

Data analysis followed the best practice guidelines of Miles and Huberman (1994), which consisted of the following steps: First, the researchers read through all included articles to get an overview of the data; noting initial ideas. Second, initial codes were generated based on features of the data which became systematically apparent when working through the data set. Third, the various codes were clustered into potential themes based on similar characteristics. Fourth, the researchers then reviewed the themes in relation to the coded extracts in order to generate a thematic map based on the frequency of occurrence. Fifth, a process of on-going analyses and constant refinement was followed in order to specify the elements of each theme and to ensure that the overall analyses tell a coherent story with clear definitions, names and labels for each theme. Finally, the themes were collated based on frequency of occurrence, used as a means to develop the definition of positive coaching and to identify the most prominent components in a positive coaching process. Here all the codes were discussed and verified amongst the research team.

## Findings

The data obtained via the systematic literature review was processed using thematic content analyses. The most frequently occurring themes relating to positive psychological coaching definitions and -models were reported separately.

### Common Elements of Positive Psychological Coaching Definitions

Table 1 summarizes and provides an overview of our findings related to the defining characteristics of positive psychological coaching. In our final selection of articles, we first extracted the information related to conceptual definitions provided by the various authors. Here the frequency of occurrence related to the amount of times a given component was mentioned within and between different records. Second, we identified the shared commonalities between those conceptualizations, which resulted in 20 commonly occurring themes.

**Table 1:** Common elements from positive psychological coaching definitions.

**Element**	**Frequency (*N* = 94)**	**Percentage**	**Quotation**	**References**
Identification, utilization, optimization and development of strengths and personal resources	19	20.21	‘It focuses on helping clients to use their existing strengths to identify vision of what they want and turn it into reality [through focusing on] strengths, vision and dreams.'	Kauffman, 2006
Facilitating personal growth, optimal functioning and enhancing wellbeing	14	14.89	‘Coaching approaches that seek to improve short term wellbeing (i.e. hedonic wellbeing) and sustainable wellbeing (i.e. eudaimonic wellbeing) using evidence-based approaches from positive psychology and the science of wellbeing and enable the person to do this in an on-going manner after coaching has completed.'	Passmore and Oades, 2014
Directed toward enhancing positive states, traits and behaviors	12	12.77	‘[…] enhancing self-regulation, insight, resilience, self-efficacy and wellbeing by facilitating the establishment and pursuit of self-concordant goals.'	Grant and Spence, 2010
Collaborative relationship between coach and client	7	7.44	‘[…] action-orientated collaborative relationship in which the coach is the facilitator.'	Freire, 2013
Actualizing client's potential	6	6.38	‘[A process that…] encouraged [individuals] to seek positive things in life, harnessing the best in people and inspiring them to live out their potential.'	Freire, 2013
Utilizing positive psychological evidence-based approaches	5	5.32	‘Coaching can learn from positive psychology about research and scientific rigor.'	Linley and Kauffman, 2007
Working with well-adjusted individuals	5	5.32	‘The client is already “whole” and skilled.'	Kauffman and Scoular, 2004
Socratic goal setting and achievement	4	4.25	‘[It] is a Socratic, future- focused, collaborative conversation between a coach and the client, during which the coach uses open questions, affirmations, reflective listening, summaries, and information exchange to stimulate and encourage self- awareness, personal responsibility, and behavioural change thought likely to lead to improved wellbeing outcomes over time.'	Anstiss and Passmore, 2017
Developing skills and capabilities	3	3.19	‘Positive psychology applied to coaching […] creates the conditions for skill and capability development beyond the usual professional activities, or beyond the prescribed area of organizational role […]'	Castiello D'Antonio, 2018
Focus on strengths not weaknesses	3	3.19	‘They focus on strengths rather than on weaknesses and use a variety of assessment tools to explore character strengths, life satisfaction, and potential routes to peak performance.'	Tarragona, 2015
Active listening	2	2.12	‘Positive psychology coaches listen for strengths and assets that a client may not be aware of, reflect back what is going right, ask questions that elicit images of better futures, and help clients define action steps supported by wellbeing theories.'	Yeager and Britton, 2017
Clients have the capacity to develop	2	2.12	‘Coach who believes in the client's ability to cope and change in positive ways, and who can identify, value, and develop the client's “muscles.”'	Noble et al., 2000
Developing a personal vision (strategy)	2	2.12	‘It focuses on helping clients to use their existing strengths to identify vision of what they want and turn it into reality [through focusing on] strengths, vision and dreams.'	Kauffman, 2006
Take ownership of growth	2	2.12	‘Taking charge of his own career development (ownership) and life-professional project.'	Castiello D'Antonio, 2018
Having a balanced view of the client's strengths and limitations	2	2.12	‘A positive psychology theoretical base does not assume that clients are paragons of virtue or that everything goes smoothly.'	Kauffman et al., 2015
Enhance professional development	2	2.12	‘It is a relationship formed between a coach and the client for the purpose of attaining professional or personal development outcomes.'	Grant and Spence, 2010
Aids in coping with work-demands	1	1.06	‘Positive psychology–based leadership coaching also paradoxically assists leaders to grapple with the inevitable negative, toxic, or near-impossible demands of business life.'	Kauffman et al., 2015
Continuous support	1	1.06	‘[The coaching process helps clients] develop and implement solutions to ongoing challenges faced during goal striving.'	Grant and Spence, 2010
Holistic approach to development	1	1.06	‘[It] is a well being intervention approach in which clients are taught strategies and skills aimed at helping them to identify, pursue, and fulfill their most cherished needs, goals, and wishes in sixteen valued areas of life said to comprise human wellbeing or happiness.'	Frisch, 2013
Short- to medium term relationship	1	1.06	‘Strengths-based coaching is a short to medium term strengths focused developmental process aimed at harnessing the inner potential of a client in order to optimise his/her performance and to actualise his/her potential.'	van Zyl et al., 2016b

In these papers, 20.21% mentioned that positive psychological coaching includes the “identification, utilization, optimization, and development of strengths and personal resources.” For example, Kauffman (2006) described positive psychological coaching as follows: “It focuses on helping clients to use their existing strengths to identify vision of what they want and turn it into reality [through focusing on] strengths, vision, and dreams.” A total of 14.89% described “facilitating personal growth, optimal functioning, and enhancing wellbeing” as core aspects of the positive psychological coaching process. Passmore and Oades (2014), for example, noted that these are “coaching approaches that seek to improve short term wellbeing (i.e., hedonic wellbeing) and sustainable wellbeing (i.e., eudaimonic wellbeing) using evidence-based approaches from positive psychology and the science of wellbeing and enable the person to do this in an on-going manner after coaching has completed.”

Approximately 7.45% described positive psychological coaching as a “collaborative relationship between coach and client.” Freire (2013) wrote that it is an “[…] action-orientated collaborative relationship in which the coach is the facilitator.” This relationship, according to 12.77%, is “directed toward enhancing positive states, traits, and behaviors,” such as “[…] enhancing self-regulation, insight, resilience, self-efficacy and wellbeing by facilitating the establishment and pursuit of self-concordant goals” (Grant and Spence, 2010). About 5.32% of articles highlighted the need for positive psychological coaching to “utilize positive psychological evidence-based approaches.” As Linley and Kauffman (2007) wrote, “coaches can learn from positive psychology about research and scientific rigor.” Another part of positive psychological coaching, mentioned by 4.26%, is “Socratic goal setting and achievement”: “[It] is a Socratic, future- focused, collaborative conversation between a coach and the client, during which the coach uses open questions, affirmations, reflective listening, summaries, and information exchange to stimulate and encourage self- awareness, personal responsibility, and behavioral change thought likely to lead to improved wellbeing outcomes over time” (Anstiss and Passmore, 2017). This would help to “actualize clients' potential” as claimed by 6.38%. Freire (2013) wrote in this regard that it is “[a process that…] encouraged [individuals] to seek positive things in life, harnessing the best in people and inspiring them to live out their potential.”

According to 3.19% of the records, positive psychological coaches place the “focus on strengths not weaknesses”: “They focus on strengths rather than on weaknesses and use a variety of assessment tools to explore character strengths, life satisfaction, and potential routes to peak performance” (Tarragona, 2015). Further, 2.13% noted the importance of “active listening.” Yeager and Britton (2017) wrote: “Positive psychology coaches listen for strengths and assets that a client may not be aware of, reflect back what is going right, ask questions that elicit images of better futures, and help clients define action steps supported by wellbeing theories.”

The goals of positive psychological coaching, as mentioned in the identified papers, included (a) to create “a personal vision (strategy),” described by 2.13%, and (b) to “develop skills and capabilities,” according to 3.19%. For example, Kauffman (2006) wrote that “it focuses on helping clients to use their existing strengths to identify vision of what they want and turn it into reality [through focusing on] strengths, vision and dreams.” Castiello D'Antonio (2018) added that “positive psychology applied to coaching […] creates the conditions for skill and capability development beyond the usual professional activities, or beyond the prescribed area of organizational role […].”

In terms of client characteristics, 5.32% of articles described that positive psychological coaches “work with well-adjusted individuals” in the sense that “the client is already ‘whole' and skilled” (Kauffman and Scoular, 2004). A further 2.13% assumed that “clients have the capacity to develop,” e.g., Noble et al. (2000) described a “coach who believes in the client's ability to cope and change in positive ways, and who can identify, value, and develop the client's ‘muscles'.”

Still, 2.13% of authors point out that coaches need to “have a balanced view of the client's strengths and limitations”: “A positive psychology theoretical base does not assume that clients are paragons of virtue or that everything goes smoothly” (Kauffman et al., 2015). After all, it is the responsibility of clients to take “ownership of their growth,” as reported by another 2.13%. The client “taking charge of his own career development (ownership) and life-professional project” is mandatory, according to Castiello D'Antonio (2018).

Four individual articles, contributing 1.06% to the final estimate, added that positive psychological coaching takes a “holistic approach to development” and provides “continuous support” during the “short- to medium term relationship” to “enhance professional development” and “aid in coping with work-demands.” Regarding the holistic approach, Frisch (2013) recorded that it “is a wellbeing intervention approach in which clients are taught strategies and skills aimed at helping them to identify, pursue, and fulfill their most cherished needs, goals, and wishes in 16 valued areas of life said to comprise human wellbeing or happiness.” The notion of continuous support is provided by Grant and Spence (2010), who said that the coaching process helps clients to “develop and implement solutions to ongoing challenges faced during goal striving.” That it is a short- to medium-term relationship was contributed by van Zyl et al. (2016a): “Strengths-based coaching is a short to medium term strengths focused developmental process aimed at harnessing the inner potential of a client in order to optimize his/her performance and to actualize his/her potential.” The emphasis on professional development was brought forward by Grant and Spence (2010), who wrote that “it is a relationship formed between a coach and the client for the purpose of attaining professional or personal development outcomes.” Lastly, Kauffman et al. (2015) add that “positive psychology–based leadership coaching also paradoxically assists leaders to grapple with the inevitable negative, toxic, or near-impossible demands of business life.”

### Common Elements of Positive Psychological Coaching Models

Next, from the 24 included articles, several common elements relating to coaching models, approaches, or frameworks were extracted. Here, the frequency of occurrence related to the number of elements which was specifically mentioned as part of a a given phase. These common elements were used as indicators for eight overarching themes which we labeled as “Coaching Phases”. These eight phases were: (1) Creating the relationship, (2) Strengths profiling and feedback, (3) Developing an ideal vision, (4) Realistic goal setting, strategizing, and execution centered around strengths, (5) Learning transfer, (6) Action tracking and evaluation, (7) Empowerment, and (8) Concluding the relationship and re-contracting. Table 2 provides a descriptive summary of the extracted themes, elements, frequencies, and supporting quotations from the papers.

**Table 2:** Common elements from positive psychological coaching models.

**Coaching phase (theme)**	**Elements**	**Frequency**	**Quotation**	**References**
Strengths profiling and feedback (*f* = 24)	Developing insight into strength use	3	‘The coach helps the staff member to appreciate the power and opportunities that his or her dominant strengths give them. This often comes by the staff member reflecting on how their strengths have already helped them to be successful or in some cases have made certain jobs more difficult. We filter the world through our strengths either knowingly or unknowingly, so this is another aspect of strengths that coaches discuss.'	Dyess et al., 2017
	Strengths diagnosis and providing feedback to client	11	‘It is therefore imperative in this phase to make the coachee aware of his/her strengths through either (a) strengths based psychometric assessments, (b) strengths-based inquiry and (c) strengths-based identification initiatives.'	van Zyl et al., 2016b
	Strengths profiling based on present/past successes	4	‘Exploring with the client the kind of activities he or she currently finds engaging, the things she or he used to find engaging but have stopped doing.'	Anstiss and Passmore, 2017
	Diagnosing quality of Life and wellbeing	4	‘Quality of Life should be tested throughout planning and evaluating the [coaching] intervention.'	Frisch, 2013
	Developing strengths and enhancing competencies	3	‘The main function of this phase is to develop the coachee's competence through strengths enhancement, building and utilisation activities. The coachee is encouraged to [develop strengths] in the current work-related reality.'	van Zyl and Stander, 2013
Realistic goal setting, strategizing, and execution centered around strengths (*f* = 22)	Establishing specific personal/work related goals and means to achieve such centered around the client's strengths	17	‘The last step in the coaching process is to help establish staff goals and determine how the staff member will invest in the development of their talents and strengths by using them more effectively in their work. Coaches help staff see how their strengths can be used to achieve their career goals by investing in activities that build on their strengths.'	Dyess et al., 2017
	Identify available resources and formulate a utilization plan	2	‘The coaching process facilitates goal attainment by helping individuals to […] identify personal resources and formulate action plans.'	Grant and Spence, 2010
	Framing solutions and action plans to address problems	3	‘This phase involves developing or framing solutions and action plans to current challenges and developmental areas. This is done through solution-building conversations.'	van Zyl et al., 2016b
Empowerment (reframing, reinforcement) (*f* = 16)	Strengthen affirmative capability	3	‘Strengthen affirmative capability to build hope and sustain momentum for ongoing positive change and high performance.'	Gordon and Gucciardi, 2011
	Reframing the victim- to a survivor mentality	3	‘Encouraging coachees to retell their stories as survivors, rather than victims. This aids in altering the coachee's perspectives of the presented problem and establishes a sense that numerous possibilities exist to understanding the problem […] which aids the coachee to shed the victim mentality.'	van Zyl and Stander, 2013
	Motivating client by highlighting strengths use to build self-efficacy	4	‘Enhance motivation by identifying strengths and building self-efficacy.'	Grant and Spence, 2010
	Empower clients	3	‘Establishing a positive connection between coach and client. Leading the client to a more empowering perspective. Affirming a sense of the possible. Cultivating and supporting the cilent's belief in a positive future.'	Gordon, 2016
	Building sustainable resilience	3	‘Build a level of resilience which will fortify the internal psychological barriers which buffer against reoccurrences in the future.'	van Zyl et al., 2016b
Creating the relationship (*f* = 16)	Establishing rapport and creating a conducive environment	12	‘In order to establish rapport, the coach needs to attend to any physical barriers which might impact or interrupt the process. The coach should create a calm and trusting environment in order to establish the perception that the coach is providing his undivided attention to the coachee. The coach should […] present genuine unconditional positive regard, free from judgement. This in turn establishes the perception that the coach is more attentive, empathic and caring. Further, a process of active listening needs to be invoked in order to show that the coach comprehends, retains and responds to what the coachee is presenting.'	van Zyl and Stander, 2013
	Clarifying expectations between all stakeholders	3	‘The purpose of this phase is to clarify the expectations between (a) the coach/coachee, (b) coach/senior management, (c) coach/direct manager and (d) coachee and his/her direct manager, in order to establish rapport, transparency of expectations and to include the organizational context (e.g. vision/mission/strategy) into the coaching process.'	van Zyl et al., 2016b
	Creating a positive relationship	1	‘Strengthen and deepen the positive relationship with the client.'	Anstiss and Passmore, 2017
Developing an ideal vision (*f* = 11)	Creating a vision of the best possible self	7	‘Help the client create a clear vision of a positive future that stretches beyond the limits of their current comfort zone and level of performance.'	White and Barnett, 2013
	Identify future-orientated desired outcomes	1	‘The coaching process facilitates goal attainment by helping individuals to identify [future-orientated] desired outcomes.'	Grant and Spence, 2010
	Identify coaching themes	3	‘The focus is to determine the coachee's areas of development through understanding the current challenges in his/her current work-related reality (van Zyl and Stander, 2013). As such, a deliberate attempt needs to be employed in order to reveal the coachee's (a) perceptions associated with current difficulties/challenges, (b) reasons why these exists, (c) the factors attributable to the challenges, (d) the possible consequences if these are unresolved and (e) the meaning derived from the given challenging context.'	Stander, 2016
Action tracking and continuous evaluation (*f* = 6)	Tracking the progress of goal achievement	2	‘[Continuously]…monitor and evaluate progression towards goal attainment.'	Grant and Spence, 2010
	Continuous assessment of wellbeing	3	‘Evaluation of the process should continue throughout the coaching intervention in order to ensure that the developmental strategy is on track.'	van Zyl and Stander, 2013
	Revisiting or modifying existing action plans	1	‘Modify action plans [when necessary].'	Grant and Spence, 2010
Learning transfer (*f* = 5)	Provide client with “Home-Work” to reinforce learnings	2	“‘Instruct” our clients about topics that are relevant to their situation, comment on evidence, share findings, or recommend a book or a video by an expert on the topic we are discussing.'	Tarragona, 2015
	Identify appropriate positive psychological self-administered interventions	2	‘Using evidence-based approaches from positive psychology and the science of wellbeing- and enable the person to do this in an on-going manner after coaching has completed.'	Passmore and Oades, 2014
	Look for opportunities for active skill development	1	‘Support coachees in handling essential but difficult emotions as well as continuing to develop methods to increase their experiences of joy, contentment and hope through active skill development.'	Sims, 2017
Concluding or re-contracting (*f* = 3)	Evaluating and re-contracting the relationship	3	‘Evaluated against the initial objectives [at the onset of the coaching process], both the coach and coachee needs to determine the success of the intervention. In the scenarios where the expectations have not been met, or if the coachee presents a need for further intervention, a re-contracting process could be initiated.'	van Zyl et al., 2016b

The results show that the most frequently occurring theme derived from the records was “*Strengths Profiling and Feedback*” (*f* = 24). The coach plays an important role in determining what the client's strengths are, what helps them to overcome hurdles, and what gives them hope (Tarragona, 2015). From this perspective, coaches employ various techniques or tools to diagnose the strengths of clients as well as to assess wellness/wellbeing and to provide feedback on the findings. Given that the client knowingly or unknowingly filters the world through his/her strengths (Dyess et al., 2017), a stronger focus needs to be placed on the diagnosis of strengths as opposed to highlighting developmental areas (Linley and Kauffman, 2007). Therefore, the coach facilitates a process to enable clients to appreciate the power and opportunities of dominant strengths (Dyess et al., 2017). Here, coaches may employ various strengths based psychometric assessments (e.g., VIA Signature Strengths Inventory), strengths-based inquiry (e.g., Strengths Based Interviewing), and strengths-based identification initiatives (e.g., Strengths Spotting or Listening for Strengths) to aid in the diagnosis of strengths (van Zyl and Stander, 2013; van Zyl et al., 2016a).

Strengths profiling initiatives, such as guided self-reflections, may also be actioned in order to explore the presence of strengths in past experiences or within the present. Clients may be asked to describe an event where they felt particularly good or at their best and reflect upon the factors (strengths) which contributed to or manifested in this experience (Anstiss and Passmore, 2017). Specifically, the client is facilitated to reflect on the function of strengths in their current realities (Linley and Kauffman, 2007). This is done in order to aid the client to develop insight into how/when their particular strengths manifest, and how to deploy such in the future (Dyess et al., 2017). Further, through becoming aware of one's strengths, clients could actively use such to develop job-related competencies (van Zyl and Stander, 2013). Finally, quality of life and wellbeing also needs to be assessed as such is an outcome of strengths use (Frisch, 2013). When individuals use their strengths, it has a direct, positive effect on their experience of overall life satisfaction/quality (Dyess et al., 2017). Therefore, tracking life satisfaction, wellbeing or quality of life would be a good indicator for the effectiveness of strength utilization (Frisch, 2013; van Zyl and Stander, 2013; Stander, 2016; van Zyl et al., 2016a).

The second most frequently occurring theme related to *Realistic Goal setting, Strategizing, and Execution centered around Strengths* (*f* = 22). Grant and Spence (2010) postulate that the purposeful pursuit of goals sits at the heart of coaching. Therefore, the focus is primarily on developing specific personal or work-related goals and determining a means to achieve such in a holistic and systematic fashion (Dyess et al., 2017). Clients are encouraged to set goals that are either aligned to their strengths or goals aimed at developing a given strength further (Spence and Grant, 2012; van Zyl and Stander, 2013). The coach must assist the client to access and develop unrecognized attributes, learn from other life experiences and set clear, specific, and tailor-made goals (Kauffman and Scoular, 2004). Here, the coach will actively clarify the purpose of the goals and aid in the development of a mutually agreed upon developmental strategy or plan (Dyess et al., 2017). This developmental plan is aimed at achieving the client's dream, affirming the client's reality, and supporting future actions (Frisch, 2013; White and Barnett, 2014). The function of the coach is to facilitate the motivation and commitment of the client to actively implement the designed developmental strategy (Frisch, 2013; Stander, 2016). Further, within this phase, clients are encouraged to identify their available and required personal resources needed in order to fast track goal achievement (Spence and Grant, 2012). Along with the developmental plan, the coach aids in constructing a personal resource map, which highlights the available/required physical, emotional, social, and fiscal resources needed to achieve their goals (van Zyl and Stander, 2013). This map is translated into a utilization plan which aligns goals, to resources (van Zyl et al., 2016b).

Another important part of establishing goals and action plans is to determine how the client will use his/her talents and strengths more effectively in their workplace (Dyess et al., 2017). Once a client realizes why competence in a particular activity is important, he/she can develop a strategy to spend more time on the activity (Linley and Kauffman, 2007). They further state that having clear goals, balancing challenges and skills, maintaining a high level of goal congruence, focusing on doing well and having immediate feedback is important in the setting of goals.

The third most frequently occurring theme or phase relates to *Empowerment* (*f* = 16). In this continuous phase clients are empowered to take ownership for their personal development (Gordon, 2016). This is not a once off phase but rather manifests in every interaction with the client and/or phase of the coaching process (van Zyl and Stander, 2013). The client's affirmation capability is strengthened in order to aid in the generation of perpetual motion needed to facilitate sustainable change and high performance (Gordon and Gucciardi, 2011). Clients need to be motivated by highlighting and reaffirming the strengths employed in given scenarios in order to build self-efficacy (Spence and Grant, 2012). The coach needs to cultivate and support clients' internal belief of a positive future and re-affirm their sense of that which is possible (Gordon, 2016). Further, clients also need to be encouraged to reframe the negative or challenging experiences of their work/private lives. Clients need to reframe these negative narratives as survivors, rather than victims, which in turn alters their perspective of the problem (van Zyl et al., 2016a). Through this process, the client is made aware that numerous possibilities exist in order to approach, interpret and experience a manifested problem (van Zyl and Stander, 2013; Stander, 2016). Through this reconditioning process, the client would systematically start shedding the victim mentality and would find it increasingly easier to look for the positive even in the most challenging environments (Stander, 2016).

The fourth most frequently occurring phase or theme was “*Creating the Relationship*” (*f* = 16) which is a function of (a) establishing rapport and creating a conducive coaching environment, (b) clarifying expectations between all stakeholders in the coaching process and (c) creating a positive relationship with the client. Dyess et al. (2017) mentioned that effective coaching begins with building a solid, positive relationship. Creating the relationship is seen as a pre-coaching phase, in which the focus is on establishing a positive, open, trusting, supporting, non-judgemental, psychologically safe, and collaborative relationship with the client (Oades et al., 2009; Stander, 2016; Dyess et al., 2017; Yeager and Britton, 2017). Most records in the sample argued that creating and maintaining sound positive relationships between the client and coach is the most important precondition for both the experience of empowerment and the facilitation of personal development (Gordon, 2016). The coach therefore needs to ensure that he/she attends to both the physical as well as non-material barriers which may affect the coaching relationship (van Zyl and Stander, 2013). Tarragona (2015) mentions the role of curiosity to explore what works well in the client's life, what they enjoy and what values are most important to the client as a means to establish rapport.

During this phase, the coach needs to develop a thorough understanding of the client's working reality and ensure that there is alignment of expectations between stakeholders. van Zyl and Stander (2013) postulate that a psychological contract needs to be established between (a) the coach/client, (b) coach/senior management, (c) coach/direct manager, and (d) client and his/her direct manager, in order to establish rapport, transparency of expectations and to include the organizational context (e.g., vision/mission/strategy) into the coaching process.

*Developing an ideal vision* (*f* = 11) of the client's future-self came out as the fifth most frequently mentioned phases of positive psychological coaching processes. The coach is responsible to aid clients in creating a clear vision of a positive future where dreams are realized, all goals are attained, potential is actualized, and they are living in accordance with their best possible self. According to Gordon (2016), the client must be encouraged to create images of possibilities, engaging the client to give voice to her/his desired future and affirming the client's dream. The coach should assist the client in creating a clear vision of a positive future, beyond the limits of their current comfort zone and level of performance (White and Barnett, 2014). Here the focus is on expanding the capacity of the client, stretching his/her limits and then supporting the client to have faith when things get tough (Gordon, 2016). Gordon and Gucciardi (2011) mention that a dream of the ideal self must be aligned to clear, measurable results and to the discovered potential of the client. The coach must encourage clients to craft a vision that is aligned to their strengths, one where life and work has meaning, where the self-actualization tendency is active and where clients can optimize their inherent potential (van Zyl and Stander, 2013). Coaches can support clients in this process by posing questions that lead to a strategic narrative that is compelling and contributes to both meaning at work (Yeager and Britton, 2017) and in life (Stander, 2016).

As opposed to the other authors, Gordon (2016), Gordon and Gucciardi (2011), and Stander (2016) provide a framework based on the Appreciative Inquiry (AI) 4-D Cycle, which could be employed to aid the client to develop this ideal vision. Here, the authors argue that strength based coaching questions could be posed to aid the client in crafting their ideal future selves, which are categorized into four phases: Phase one is about Discovering strengths and the meaning of strengths from the perspective of the client. Then the client is encouraged to Dream about the ideal state and what it means to him/her on a practical level. In the Design phase the client must develop an action plan to achieve the ideal state while the focus of the last phase, Destiny, is to build hope and sustain momentum for ongoing positive change and high performance. From this ideal vision and analyzing the gap between the current and desired state, specific coaching themes can be derived, which can form the basis of the coaching relationship (Stander, 2016).

*Action Tracking and Continuous Evaluation* was mentioned as an important component of the coaching process by a number of authors (*f* = 6). The focus is on constantly evaluating the progress and effectiveness of the coaching intervention, the extent toward which goals are achieved and gains maintained, as well as a continued assessment of the wellness and wellbeing of the client (Gordon and Gucciardi, 2011; van Zyl and Stander, 2013). Linley and Kauffman (2007) highlighted the importance of continuous evaluation as changes in the aspirations, goals, and needs of clients may change throughout the coaching relationship. Therefore, goals and aspirations need to be continuously revisited and modified based on the current reality of the client (Grant and Spence, 2010).

The penultimate coaching phase/theme extracted from the records was *Learning Transfer* (*f* = 5). Some authors mentioned that personal growth and development is a continuing process, which stretches beyond the confines of the coaching session (Tarragona, 2015). Learnings from the coaching sessions need to be transferred to the work environment in order to aid the client to practice the skills learned during coaching and to take ownership of the learning process (Passmore and Oades, 2014; Sims, 2017). Providing clients with “homework” between sessions increases engagement with and adherence to the coaching process (Frisch, 2013), whilst it provides an active means (in a safe environment) to develop and grow (Tarragona, 2015). Tarragona (2015) argued that the role of the coach in this phase is to identify positive psychological topics that are relevant for the client, to share learnings and to recommend resources (such as books and videos) to facilitate self-development. These resources should reinforce or expand on what was facilitated in the coaching sessions. Further, the coach should identify appropriate positive psychological self-administered activities aligned to the strengths of the client, in order to ensure that significant gains in the client's positive state are facilitated (e.g., the gratitude visit; van Zyl et al., 2016a,b). These evidence-based positive psychological practices need to be aligned to the content of a given coaching session, they needs to be challenging (though not demanding), there should be an opportunity to actively practice such at work (van Zyl et al., 2016b) and they should be aimed at the active development of skills (Sims, 2017). The client should be requested to develop a portfolio of evidence while practicing these activities and developing their skills (Stander, 2016).

The final phase derived from the records was *Concluding and Re-Contracting* (*f* = 3) the coaching process. van Zyl and Stander (2013) believed that during this phase the client and the coach should formatively assess whether the coaching processes yielded the desired results and either prepare the client for the termination of the relationship or if there is a need for further development, a process of re-contracting could be initiated (van Zyl and Stander, 2013). Here, all stakeholders (clients, managers, HR, and the coach) should honor the progress made by the client and the coach is responsible to determine whether the changes that took place were a direct result of the positive psychological coaching process. Finally, Stander (2016) argued that at the end of the coaching process, the coach needs to calculate the return on investment of such for the company.

## Discussion

The purpose of this systematic review was to clarify the theoretical conceptualization of positive psychological coaching and determine the common components of positive psychological coaching models/frameworks. Specifically, the aim was to construct a commonly shared understanding or “definition” of the concept, and to construct a clear demarcated positive psychological coaching model/process. The initial literature search yielded 2,252 records, and through a systematic process of elimination, based on predefined inclusion/exclusion criteria, 24 academic peer-reviewed publications on positive coaching were included and coded for analyses. Data relating to definitions, and models were extracted and processed through thematic content analysis. The results highlighted 20 common elements of positive psychological coaching definitions which were used to construct an integrated definition. Further, eight commonly occurring critical components/phases of a positive psychological coaching model were found underpinning people development.

### Defining Positive Psychological Coaching

The results showed that *positive psychological coaching* can be defined as:

‘*A short- to medium-term professional, collaborative relationship between a client and coach, aimed at the identification, utilization, optimisation and development of personal/psychological strengths and resources in order to enhance positive states, traits and behaviours. Utilizing Socratic goal setting and positive psychological evidence-based approaches facilitate personal/professional growth, optimal functioning, enhanced wellbeing, the actualization of people's potential and aid in coping with work-demands.'*

Our definition of positive psychological coaching is in line with the definition by Linley and Joseph (2004) in the sense that they too argue that an aim of the positive coaching process is the promotion of optimal functioning. Furthermore, our definition echoes the one provided by van Zyl and Stander (2013) regarding the description of the coach-client relationship as professional and aimed at the identification, utilization and optimization of strengths to facilitate individual development. However, in contrast to van Zyl and Stander (2013), our definition does not specifically include positive outcomes for the organization. Our definition is further aligned with Grant et al. (2010) because they describe positive coaching as a collaborative approach applying personal goal setting and capitalizing on individual strengths. Similarly, in line with Castiello D'Antonio (2018), we highlight in our definition that positive psychological coaching is a short- to medium-term approach, aimed at enhancing positive states, such as life satisfaction and flourishing, by applying evidence-based positive psychological approaches.

In contrast, our definition deviates from Denison and Avner (2011), who described positive psychological coaching as a set of easy to use, behavioral guidelines according to best practice guidelines. While we also include in our definition that the positive psychological coaching approach should follow best practice guidelines in the form of employing evidence-based approaches, our definition focuses on the individual identification, utilization, and optimization of personal strength of the specific client. It is therefore a more personalized process rather than following mechanistic guidelines. Similarly, Orem et al. (2007) focused their definition on the development of self-compassion through an appreciative relationship with a coach. While the positive relationship with the coach is also part of our definition, our definition takes a broader focus on positive states, traits and behaviors, of which self-compassion may be part. In sum, our definition is aligned with the majority of previous definitions, though takes a broader approach and combines elements from different definitions.

Further, the results highlight that the positive psychological coaching definition or process is underpinned by eight *core principles*. *First, coaches employing a positive psychological coaching process work with relatively well-adjusted individuals*, devoid of severe psychopathology. Green and Palmer (2018) argued that the coaching relationship is facilitative, and solution-focused in nature, with the direct aim of enhancing personal fulfillment and growth. This in turn requires clients to be relatively devoid of severe psychopathology (van Zyl and Stander, 2013). Clients who present with psychopathological problems (e.g., depression or anxiety) would not gain much benefit from the coaching process and need to be referred to counseling or therapy (Seligman, 2012). Counseling or therapy is problem-focused and aimed at diagnosing psychopathological illnesses and restoring (as opposed to optimizing) psychological functioning (Green and Palmer, 2018).

*Second, the focus is on the development and optimization of personal strengths and not “fixing” weaknesses*. The positive psychological coaching process is focused on aiding clients to become aware of, utilize and develop their unique psychological strengths to reach personal goals (Kauffman, 2006; Linley and Kauffman, 2007; Seligman, 2012; Gordon, 2016) and not to focus on weaknesses (van Zyl and Stander, 2013). Positive psychological coaching is aimed at harnessing the best in people and to optimize their potential through strengths-based initiatives (Freire, 2013). When focusing on “correcting” weaknesses, the process then reinforces low expectations, creates dependency on outside resources and discourages optimal development (Seligman, 2012; Stander and van Zyl, 2019).

*Third*, despite the focus being on strengths, the *coach should have a balanced view of the client's strengths and developmental areas*. Kauffman et al. (2015) cautions against assuming that clients are solely formed out of strengths and that the coach should adopt a balanced view of strengths and developmental areas. Positive psychological coaches should aid clients to determine their developmental areas but utilize their strengths in order to address such (Gordon, 2016). However, a one-to-one ratio of “strengths” and “developmental areas” could have a negative impact on the effectiveness of the coaching process. Fredrickson and Losada (2005) proposed that a balance be struck between positive and “negative” factors during such a process, suggesting a critical ratio of three positives (strengths) to one negative (developmental area).

*Fourth, a holistic approach towards development is employed*, where the client works on matters in all domains of his/her life; capitalizing on the resources within each of the systems in which he/she function. Clients function within an eco-system of inter-related sub-systems (e.g., work, family etc.) and developing skills and capabilities only in one domain would deter from full actualization of the client's potential (Hawkins and Turner, 2019). A holistic approach toward development needs to be employed covering all valued aspects of a client's life and capitalizing on all the internal (e.g., personal resource) and external resources (e.g., social support networks) in order to enhance their wellbeing and help deal with life's challenges (Frisch, 2013; Dyess et al., 2017).

*Fifth*, the approach assumes that *clients have an inherent capacity to grow and develop*. A fundamental principle to the positive psychological coaching process is the believe that clients have an expanding ability to develop and grow through deliberate or purposeful endeavors (Noble et al., 2000; Dweck, 2009). Clients have an inherent need and capacity to develop through active effort, dedication, deliberate practice and hard work (Gordon, 2016; Purdie, 2017). Adopting such a “growth mindset” leads to clients taking on more challenges, bounce back from setbacks at a faster pace and positively affects their level of work-related performance (Palmer and Green, 2018). In effect, clients need to be made aware that they have direct control over both their development and their lives (Dweck, 2009).

*Sixth*, the *client needs to be empowered to take ownership of their own growth process*. Clients need to be facilitated in order to take responsibility for their own developmental journeys as they are not passive consumers of a product/service, but rather an active participant in the process (Noble et al., 2000). The coach should provide support and aid in discovering resources in order to aid the client to achieve their goals, but the process is fundamentally built around the extent toward which the client takes ownership of their growth process (Stander, 2016; Castiello D'Antonio, 2018).

*Seventh*, the *developmental process is based on a personal vision of the ideal self, which is translated into specific goals and actions*. The developmental process is based on a clearly defined vision of the “preferred future self” which is translated into specific, measurable, actionable, realistic, and time bound goals. Clients need to be encouraged to create clear images of possibilities, and to identify their preferred or “best possible self/future” (Gordon, 2016). The role of the coach in this process is to aid the client to develop a clear picture of the better future and to formalize such in a constructive manner (Yeager and Britton, 2017).

*Eight, the coach's role is to listen actively and to provide continuous support throughout the developmental journey*. Active listening is one of the core competencies of any type coaching process, but according to Yeager and Britton (2017), within the positive psychological coaching process, deeper level of listening is needed where the coach must listen for strengths that a client is not yet aware of. The role of the coach is to actively encourage, empower and support the client throughout the developmental journey (Green and Palmer, 2018).

### A Positive Psychological Coaching Model

**Figure 2:**
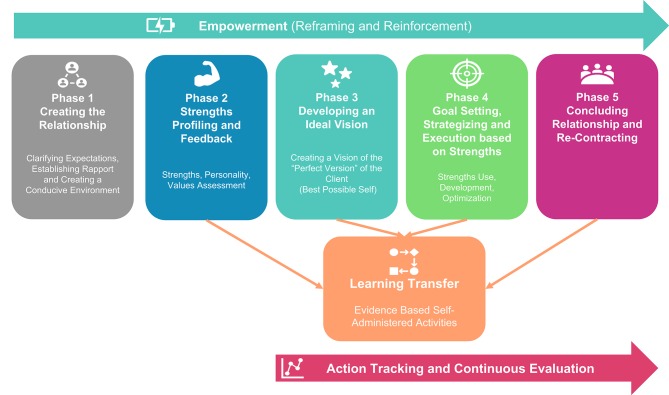
The positive psychological coaching model.

The final goal of this paper was to determine the common elements of positive psychological coaching models/approaches/frameworks in an attempt to construct a clear demarcated positive psychological coaching model or process. From the final 24 articles retained, five common sequential positive psychological coaching phases were identified: (1) creating the relationship, (2) strengths profiling and feedback, (3) developing a personal vision of the ideal self, (4) setting-, strategizing-, and executing realistic goals based around one's strengths, and lastly (5) concluding the relationship and re-contracting. These phases are supported by three continuous processes, namely (6) transferring learning to the workplace (homework), (7) empowering clients through reframing and positive reinforcement, and (8) setting clear evaluation criteria and continuously tracking actions and the developmental process. Based on these elements, a deductive approach was employed to construct a positive psychological coaching model (PPCM: c.f. Figure 2) and to arrange its phases in a logical-, chronological-, and sequential order^3^. Table 3 provides an overview of the PPCM phases, coupled with a brief description of each phase.

**Table 3:** Positive psychological coaching phases and brief description.

**Phase**	**Logical order**	**Brief description**
Phase 1	Creating the relationship	The purpose of this phase is to establish a positive, open, trusting, supportive, non-judgemental and collaborative relationship with the client. This is done through establishing rapport and clarifying expectations between the client and other stakeholders (direct line manager, and the coach). In this phase the coach needs to develop a thorough understanding of the client's environment.
Phase 2	Strengths profiling and feedback	The purpose of this phase is to aid the client to explicitly identify, develop insight into and facilitate the use/development of his/her strengths. This is done through employing strengths diagnostic tools, -interviews or techniques to identify strengths and to provide active, and constructive feedback. Wellness and Quality of Life needs to be assessed to track overall effectiveness of the coaching intervention.
Phase 3	Developing ideal vision	The client develops a clear picture of the perfect version of him/herself in the future. It can be described as an ideal future state that will stretch the client in a process to optimize his/her potential.
Phase 4	Realistic goal setting, strategizing, and execution centered around strengths	Clients need to set specific, measurable, attainable, realistic, and time bound goals that are aligned to their strengths and that builds up to their ideal vision. These goals need to be translated into a clear strategic-operational plan, which needs to be easily implementable.
Phase 5	Concluding or re-contracting	At the end of the coaching process, the effectiveness of the intervention needs to be assessed. Clients need to evaluate if goals were achieved and if so, he/she needs to be prepared for terminations. If goals were not achieved, new goals can be set, and the coaching relationship can be re-negotiated.
Continuous process 1	Learning transfer	Learning from the coaching process should be transferred to the work environment while the client takes ownership of the learning process. The focus is on actively transferring or “practicing” learnings from the coaching process in real world scenarios. Positive psychological evidence-based intervention strategies are selected that are aligned to the strengths of the client in order to maximize the efficiency and benefits associated with deliberate practice.
Continuous process 2	Action tracking and continuous evaluation	The purpose is to determine how success of the coaching process will be measured and to develop a means to actively track the effectiveness of the intervention. Both goal achievement and wellbeing are actively monitored and tracked.
Continuous process 3	Empowerment (reframing, reinforcement)	The purpose of this phase is to aid the client to feel connected to the proverbial bigger picture, reaffirm their confidence in their abilities, to aid the client to experience a sense of control over initiating and regulating behavior and to make the client feel like they are making a difference in their context. The focus is on reframing challenges as opportunities, and to find the positive in negative experiences. The coach focuses on what went “right” rather than on what went “wrong” in order to empower the client to take ownership for his/her personal development.

*Phase 1* of the PPCM relates to “*creating the relationship*.” This is a pre-coaching phase where the focus is on building a positive, open, trusting, supportive, non-judgemental, and collaborative relationship between the coach and the client (van Zyl et al., 2016a; Dyess et al., 2017). This implies that rapport must be established, clear expectations are set between all stakeholders, that the coach has deep knowledge of the client's context/environment, and that an environment is created, which is conducive to the optimization of the client's potential (van Zyl et al., 2016a).

In this phase, the coach attempts to establish rapport through attending to the physical and psychological barriers which may influence the establishment of a positive relationship conducive to the developmental process (Jorgensen et al., 2016; Jorgensen-Graupner and van Zyl, 2019). The coach attempts to explore the reality of the client through a process of active listening (Jorgensen et al., 2016) and positively infused language (Linley et al., 2009). Through active, constructive and positive communication, the coach communicates empathy, positive regard and authenticity which in turn creates a psychologically safe environment where clients can access their deepest thoughts, facilitate the development of self-insight and aids in creating an empowering environment conducive to change (Scheel et al., 2013). Both Rogers (1951) as well as Gallagher and Bennett (2018) argued that the success of a one-on-one developmental intervention largely depends on the strength of the relationship between the client and the coach, and therefore creating and maintaining a positive relationship should be a central focus during the coaching process.

Further, the coach needs to clarify expectations with the client's direct line manager as well as with senior management. This is done in order to determine (a) the potential (mis)alignment between the client's strengths and the organizational demands and (b) how the client fits into the proverbial dream/vision of the organization (van Zyl et al., 2016a). The coach also acts as a facilitator for the clarification of expectations between the client and his/her direct line manager (Stander, 2016). This is not only done in order to ensure complete alignment of the coaching process to the operational role of the client, but also to ensure that work-related expectations are clear from the onset of the process (Odendaal and le Roux, 2016). Thereafter, the coach needs to clarify expectations with the client (van Zyl and Stander, 2013). Here the focus should be on understanding the client's expectations of both the coach as well as the coaching process (van Zyl et al., 2016a). The coach also has an opportunity to clarify his/her expectations of the client/coaching process (Stander, 2016; Knotek et al., 2019). This process of expectation clarification culminates in a psychological contract, which defines the trajectory of the coaching relationship and demarcates the boundaries thereof (McComb, 2009; Hagen and Williams, 2019). This psychological contract is employed to define the beliefs, assumptions, obligations and expectations the client has about the coaching relationship and to clarify uncertainty (McComb, 2009).

Once this process has been completed, the coach needs to develop a thorough understanding of the client's environment (Knotek et al., 2019). The coach needs to explore the nature of the client's role within the organization, his primary function, the contextual challenges the client faces and the available resources at hand (Malinga et al., 2019). This is done in order to embed and align the coaching process to the organizational context (Fox, 2015). Upon completion of this phase, the coach and client can move on to the next.

*Phase 2* in the PPCM aims to subject the client to a “*strengths profiling and feedback*” process. The focus here is to explicitly identify or diagnose the psychological strengths of the client through psychometric assessments (e.g., VIA Signature Strengths Inventory, Realise2, Strengths Finder 2.0), fit-for-purpose simulations (e.g., Talent Development Centres), strengths-based interviewing, strengths-spotting or other strengths-exploration exercises (Stander and van Zyl, 2019; van Zyl and Rothmann, 2019a). The client needs to become aware of his/her strengths and develop insight as to how these could be used to foster personal development and achieve goals (McQuaid et al., 2018). This is done through providing strengths-based feedback, focusing on which strengths are more likely to manifest in the day-to-day life of the client (Burke and Passmore, 2019). Specific focus needs to be placed on the development of strengths, and how such could be used to enhance competence in various applied domains (Stander, 2016). This is done to aid clients to overcome their preoccupation with developmental areas. Focusing and continually highlighting developmental areas reinforces low expectations, creates dependency on outside resources (such as the coach) and discourages optimal development (van Zyl and Rothmann, 2019b). Finally, the coach needs to also aid the client to identify the availability of potential personal resources and diagnose quality of life/wellbeing in order to identify potential coaching themes (Palmer and Green, 2018).

*Phase 3*, of the PPCM aims to aid clients in “*developing an ideal vision*” of themselves. During this phase, the coach engages in a collaborative strengths-based inquiry process in order to aid the client to (a) discover the best of his/her current situation, (b) imagine what his/her life could be, and (c) to develop a clear and compelling vision of the future where incentives, persuasion, or coercion is not needed (Whitney and Trosten-Bloom, 2010; White and Barnett, 2014). In effect, the client must develop a clear picture of the “perfect”- or best-possible version of him/herself in the future. It can be described as an ideal future state that will stretch the client's boundaries in a process to optimize his/her potential (Gordon and Gucciardi, 2011). Based on insights from the previous phase, the coach should facilitate clients to craft this vision around their desires and not around what they believe their lives “should” be like. This vision should be crafted around the best possible versions of themselves, where dreams are realized, all goals are attained, potential is actualized, and they are living in accordance with their best possible self (Palmer and Green, 2018). Compared to traditional coaching models, with a focus on addressing problems or development needs, the PPCM is focused on what is the best (ideal) state for the client and determining how this state could effectively be achieved through utilizing strengths. According to Gordon (2016) and Gordon and Gucciardi (2011), Appreciative Inquiry could be used as a framework for developing such an ideal vision. From this perspective, clients are facilitated to discover what is currently working well, to dream about a positive future, to align the dream to the practicalities of the current reality and to aid in the execution there of. From this, a clear action plan can be developed and implemented in order to aid the client to strive toward this ideal vision.

Phase 4, “*goal setting, strategizing, and execution*,”^4^ is common to most coaching models or frameworks. In this phase, the information of the previous phases is integrated into a solutions-building conversation in order to (a) set goals, (b) develop an implementation strategy, and (c) facilitate the execution thereof. During *goal setting*, the client needs to perform a gap analysis through contrasting his/her current reality with the ideal vision crafted in the previous phase in order to determine the specific action step required to close these proverbial gaps (McQuaid et al., 2018; Stander and van Zyl, 2019). This can be done through various evidence-based techniques, such as using the Wheel of Life Brainstorming Framework (Byrne, 2005), or the Five Paths to Happiness or CASIO exercise (Frisch, 2013) to determine the discrepancy between the current and desired end state. This can then be translated into specific, measurable, attainable, relevant, time-bound, evaluated and rewarded (SMART) goals, which draw from the strengths and positive capacities of the client, in order to close the aforementioned gaps (Hyatt, 2018). Furthermore, there needs to be an active balance between the challenges the clients set for themselves, their current level of skill and their capacity to activate their strengths (Seligman, 2012). If the challenges far exceed the capabilities of the client, it would have negative consequences on the health, wellbeing, motivation, and performance of the client (Seligman, 2012). Therefore, careful consideration needs to be taken when setting goals. Cole and Stavros (2019) suggested that a positive strengths-based approach should be used to manage such. They argued that clients use the SOAR framework to determine and translate the ideal vision into actionable goals through focusing on identifying the strengths they could use-, the opportunities available to aid in-, the aspirations they have from- and results associated with each goal they want to achieve.

Based on these goals, the client and coach need *to develop an implementation strategy* for how these goals could be achieved. Here, meta-goals should be broken down into smaller, more digestible actions with clear time frames and actions (Stander, 2016). The client needs to identify the resources and strengths needed in order to ensure that the strategy is realistic and easily implementable (Gordon, 2016). Further, clients should also be facilitated to look for their proverbial “blind spots” (factors which might influence the goal achievement process) (Tarragona, 2015). Once these goals have been translated into easily implementable strategies, the client needs to find supporters/mentors who could aid them in maintaining momentum once strategies are being actioned. Further, clear deadlines need to be set, and the client needs to commit to and be accountable for their achievement (van Zyl et al., 2016a). This strategy should be captured in an individual development plan, which highlights the goals, the developmental actions, the resources required and the timelines relating to its achievement.

Finally, the client needs to be empowered to *execute the developed strategy*. Here the client needs clear, immediate and developmental feedback on each stage of the process. Realtime updates on goal progress will facilitate more commitment and accountability (Stander and van Zyl, 2019). During execution, a high level of congruence between the goals, the strategy and strengths should exist.

The chronological phases of the PPCM culminates in Phase 5, “*concluding the relationship and re-contracting.”* At the end of the coaching process, the effectiveness of the coaching intervention needs to be formally assessed and the client prepared for termination or re-contracting of the relationship (van Zyl and Stander, 2013). Clients need to evaluate if goals were achieved and if so, need to be prepared for termination of the relationship. Coaches could evaluate the client's progress through conducting psychometric assessments, positive 360-degree evaluations and the like, and through comparing such with the original results at the start of the coaching process (Stander, 2016). The changes over time would act as indicators of progress/development. Similarly, clients, managers, HR, and the coach should reflect upon the progress made by the client and the victories celebrated. If goals were not achieved, new goals can be set, and the coaching relationship can be re-negotiated. The focus here is on determining if the gaps identified at the start of the coaching process was closed (van Zyl and Stander, 2013).

Although the PPCM has a clear start and end date, the development process in itself is not linear. Given the fast-changing and ambiguous business environments in which clients function, short-term or unexpected changes in client's roles, or the demands of the business, could result in the re-formulation of the client's goals or result in reprioritization thereof. As such the coaching model should be flexible to adapt to situational demands or needs as it may occur. The preceding sequential, chronological phases are supported by three *dynamic or “continuous processes*” that strengthens the interaction between the different phases. These three continuous processes apply to all the phases of the coaching model, but at the same time build on and are supported by each other.

First, Continuous Process 1 relates to “*learning transfer.”* Learnings occurring from the coaching process should be transferred to the work environment in order to aid the client to both practice these skills in a real life setting and to also be empowered to take ownership of the learning process (van Zyl et al., 2019). The coach can provide “homework” to aid the client in developing new skills and capabilities or to ensure engagement with and adherence to the coaching process (Hayes and van Zyl, 2019). The client should engage in deliberate practices, focusing on using strengths in a constructive and developmental way (Passmore and Oades, 2015). The activities selected by the coach to support the developmental process need to be evidence-based and should strongly draw from the positive psychological intervention literature (Fox, 2015). Further, clients need to be empowered to introduce these evidence-based practices or skills in real world settings and be made aware that obstacles or failures to do so should be seen as a learning opportunity (Kauffman, 2006; Stander, 2016). This process is facilitated from Phase 2 of the coaching process, right through to the final session.

Continuous Process 2 relates to “*action tracking and continuous evaluation*.” Here the focus is on the continuous evaluation of the coaching process, goal-achievement, and openness for changing aspirations of the client. Tracking the developmental process aids in ensuring that the developmental initiatives are in effect aiding the client to achieve his/her goals and to intervene if evidence suggests that the client is not on track with his/her goal achievement (van Zyl and Stander, 2013). At the onset of the coaching process, stakeholders need to develop clear, measurable criteria that constitutes “success” within the coaching process (van Zyl et al., 2016a,b). Based on these criteria, the coach and client need to develop a means through which to track actions and goal achievement. This could take the form of weekly updates, online coaching tracking software or quantitative assessments of wellbeing or performance (Linley et al., 2009; Stander and van Zyl, 2019). It is imperative that both goal achievement and wellbeing is assessed on a monthly basis in order to ensure that the developmental trajectory is on track (Noble et al., 2000; van Zyl et al., 2016a) and that it does not have any negative consequences for the wellbeing of the client (Gordon and Gucciardi, 2011; Frisch, 2013). Should changes in the developmental trajectory occur, the coach and client need to actively intervene or re-prioritize goals in order to ensure goal achievement is still on target (Stander, 2016). A further essential part of the evaluation process and action tracking is to determine to what extend learning is actively transferred between the coaching process and the workplace (van Zyl and Stander, 2016). To succeed in transferring learning, the client needs to be empowered to feel competent and in control of the development process. As such, the individual development plan should therefore be updated with all this information on a monthly basis.

Lastly, Continuous Process 3 relates to actions and behaviors exhibited by the coach relating to the “*empowerment*” of clients to take ownership for personal development and wellness. The purpose of this continuous process is to ensure that the client feels connected to the bigger picture and to reaffirm his/her confidence in his/her abilities. This is done to aid the client to experience a sense of control over initiating- and regulating behavior and to make the client feel like they are making a difference in their context (Kauffman et al., 2015). The coach frames questions in a positive and empowering manner in order to explore what is working well as opposed to an overemphasis on what is “wrong” (Whitney and Trosten-Bloom, 2010). Specifically, positive affirmations and positively infused questions should be used to affirm clients' faith in their own strengths and to positively reframe the world and its challenges (Grant and Spence, 2010; Yeager and Britton, 2017). The focus here is to aid the client to reframe challenges as opportunities, and to find the positive in negative experiences (Stander, 2016). Primarily, the coach should aid the client to tell stories from a survivor-, rather than from a victim-orientated position (van Zyl et al., 2016a). This aids in reframing the victim mentality and builds an internal locus of control. This empowers the client to take ownership for his/her personal development and reduces the dependency on external rewards/recognition systems (Yeager and Britton, 2017). This continuous process is a fundamental activating condition to facilitate change and is applicable to each of the five chronological phases and supports both learning transfer and the evaluating processes.

## Limitations and Recommendations

Despite thorough attempts to ensure the relevance and rigor of this systematic review, there are a number of limitations present. First, given the subjective nature of the development of the search protocol, bias could occur at any phase of the data extraction, analyses, and interpretation process. Although various processes were put into place to manage inherent biases (strict inclusion/exclusion criteria, multiple raters, calculating inter-rater reliability, conducting multiple searches with the same key words etc.), it is possible that such biases affected the process. Secondly, only academic peer-reviewed publications were included as part of the search protocol. Although this, in itself, would not be a problem within traditional systematic reviews, but within the field of positive psychology, many “popular psychology” books, practitioner-focused magazines, and the like get published daily. Although these manuscripts do not comply to the academic standards associated with the scientific method, most of these become highly cited within the academic literature due to their popularity (e.g., Biswas-Diener and Dean, 2007). In these types of manuscripts, authors have more freedom to explore and present ideas, and this is often where innovative ideas are born. Excluding popular psychology texts/books may present with a biased view. Thirdly, gray literature such as conference proceedings and those publications which were not in English were also excluded. As such, our review is limited by the potential for reporting bias. Fourthly, although thorough attempts have been made to ensure that all the appropriate literature was included, there is a possibility that a number of important publications may have been excluded either based on title, or abstracts or based on the selected keywords. Finally, the chronological ordering of the coaching phases was based on subjective and deductive reasoning processes (which were informed by the literature and the authors practical experience as coaches). This could imply that the sequential- or chronological order of the coaching phases could be in a different order.

These limitations do, however, provide an opportunity for future research. Future research should aim to contrast positive psychological coaching models and approaches presented within the popular literature with those found in this systematic review. Further, given the extensive discussion on the components of the model, and the techniques employed, future research could directly implement such and evaluate its effectiveness as a developmental framework. Interpretative phenomenological analyses could be used to deconstruct and reconstruct the coaching model in future studies in order to provide more systematic evidence for the chronological order of the coaching model.

Moreover, future endeavors in conducting research on and practicing PPC should take into account the advancements brought into the field by the “second wave positive psychology (PP2.0)” (Wong, 2011). This approach builds on the critique of positive psychology with regard to an overemphasis on the positive experiences and ideal visions, and more strongly endorses a holistic and integrative view on both the positive as well as the negative aspects of the human existence (Lomas and Ivtzan, 2016). In this regard, our positive psychological coaching model highlights the positive reframing of negative experiences and changing a victim mentality as parts of a continuous “empowerment” process. However, our and other current positive psychological coaching models may benefit from a further elaboration of concrete techniques and tools to capture more comprehensively the complexity of human life.

## Conclusion

This review provides a first attempt to systematically and scientifically consolidate the available literature on positive psychological coaching. Based on the literature, a clear and holistic definition could be derived as well as an integrative, multiphase positive psychological coaching model developed. For the academic, this article should contribute to the development of PPC as a science; for the practitioner it provides a practical framework from which to practically develop people. We are excited by the opportunities it will create for other researchers to explore, validate, critically debate, and build on this body of knowledge, enhancing the scientific image and identity of PPC. Academic institutions could structure their training programs according to the findings in this study, while for the practitioner it creates a practical and structured guide that facilitates and encourages the application of positive psychological principles in the development process of clients.

## Data Availability Statement

All records derived from the literature and employed for this study are available upon request from the corresponding author.

## Author Contributions

All authors listed have made a substantial, direct and intellectual contribution to the work, and approved it for publication.

## Conflict of Interest

The authors declare that the research was conducted in the absence of any commercial or financial relationships that could be construed as a potential conflict of interest.

## Footnotes

^1^The words “client” and “coachee” are used interchangeably in the paper.

^2^2000 marked the start of the positive psychology paradigm. Therefore, this starting point for the literature search seemed most appropriate.

^3^No article within the sample specifically stipulated a proposed length or duration of a positive psychological coaching process nor the extent of time spent of various components or phases associated with such. However, in a meta-analyses by Theeboom et al. (2014) they found that five or more coaching sessions of at least 45 min spread across 6 months are beneficial for developing coping strategies and for goal-directed self-regulation activities, four or less is required work/career attitudes, or enhancing performance/specific skills.

^4^It should be noted that although gaps between current and desired end-state are identified in this phase, and strategies developed to close such, gaps could also be identified consistently throughout the entire coaching relationship as a result of the continuous evaluation and action tracking process discussed below.
