# Supplementary material for: Positive Psychological Coaching Definitions and Models: A Systematic Literature Review
Source: Front Psychol. 2020 May 6;11:793. doi: 10.3389/fpsyg.2020.00793 (PMC7218139; doi:10.3389/fpsyg.2020.00793)
Supplement: Supplementary file 1 [file Data_Sheet_1.docx]

**Appendix A.** *Articles Included for Qualitative Synthesis*

| **Authors** | **Title of Paper** | **Journal/Book** | **Purpose** | **Application Domain** | **Citation** |
| --- | --- | --- | --- | --- | --- |
| T. Anstiss and J. Passmore | Wellbeing coaching | The Routledge companion to wellbeing at work | To combine insights from the science of wellbeing and positive psychology with coaching, to help coaches become as effective as possible in helping their clients take steps to protect and improve their wellbeing. | Organisational Contexts (General) | Anstiss, T., & Passmore, J. (2017). *Wellbeing coaching.* In C. Cooper & M. Leiter (Eds.), The Routledge companion to wellbeing at work (pp. 237-248). London, UK: Routledge. |
| A. Castiello D'Antonio | Coaching psychology and positive psychology in work and organizational psychology | The Psychologist-Manager Journal | To examine the contribution of positive psychology to coaching psychology in the organizational contexts. | Organisational Contexts (General) | Castiello D'Antonio, A. (2018). Coaching psychology and positive psychology in work and organizational psychology. *The Psychologist-Manager Journal, 21*(2), 130-150. |
| S. M. Dyess, R. Sherman, A. Opalinski and T. Eggenberger | Structured coaching programs to develop staff | The Journal of Continuing Education in Nursing | To reflect on three coaching programs: Gallup Strengths-Based Coaching, Dartmouth Microsystem Coaching, and Health and Wellness Nurse Coaching | Organisational Contexts (Nursing) | Dyess, S. M., Sherman, R., Opalinski, A., & Eggenberger, T. (2017). Structured coaching programs to develop staff. *The Journal of Continuing Education in Nursing, 48*(8), 373-378. |
| T. Freire | Positive psychology approaches | The Wiley-Blackwell Handbook of the Psychology of Coaching and Mentoring | To discuss how positive psychology science can be applied to coaching conceptualization and practices | General Life | Freire, T. (2013). Positive psychology approaches. In J. Passmore, D. Peterson, & T. Freire (Eds.), *The Wiley-Blackwell Handbook of the Psychology of Coaching and Mentoring* (pp. 426–442). West Sussex: Wiley-Blackwell. |
| M.B. Frisch | Evidence-based wellbeing/positive psychology assessment and intervention with quality of life therapy and coaching and the Quality of Life Inventory (QOLI) | Social Indicators Research | To describe Quality of Life Therapy and Coaching and review developments and research since the publication of the manual in 2006 | General Life | Frisch, M. B. (2013). Evidence-based wellbeing/positive psychology assessment and intervention with quality of life therapy and coaching and the Quality of Life Inventory (QOLI). *Social Indicators Research, 114*(2), 193-227. |
| S. Gordon | Strengths-based coaching: Case of mental toughness | Sports and athletics preparation, performance, and psychology. The psychology of effective coaching and management | To illustrate how a strengths-based approach can be used to coach behaviours | Professional Sports | Gordon, S. (2016). Strengths-based coaching: Case of mental toughness. In P. A. Davis (Ed.), *Sports and athletics preparation, performance, and psychology. The psychology of effective coaching and management* (pp. 267–283). Hauppauge, New York: Nova Science Publishers. |
| S. Gordon and D.F. Gucciardi | A strengths-based approach to coaching mental toughness | Journal of Sport Psychology in Action | To describe the history of the strengths-based approach and how to translate it into practice | Professional Sports | Gordon, S., & Gucciardi, D. F. (2011). A strengths-based approach to coaching mental toughness. *Journal of Sport Psychology in Action, 2*(3), 143-155. |
| A.M. Grant and G.B. Spence | Using coaching and positive psychology to promote a flourishing workforce: A model of goal-striving and mental health | Oxford Handbook of positive psychology and work | To outline a range of coaching applications that are frequently used in the workplace and discusses differences | Organisational Contexts (General) | Grant, A.M., & Spence, G.B. (2010). Using coaching and positive psychology to promote a flourishing workforce: A model of goal-striving and mental health. In P.A. Linley, S. Harrington, & N. Page (Eds.), *Oxford Handbook of positive psychology and work* (pp. 175-188). Oxford: Oxford University Press. |
| C. Kauffman | Positive Psychology: The Science at the Heart of Coaching | Evidence based coaching handbook: Putting best practices to work for your clients | To present a number of emerging trends in positive psychology theory and research and explore their applications to coaching | General Life | Kauffman, C. (2006). Positive Psychology: The Science at the Heart of Coaching. In D. R. Stober & A. M. Grant (Eds.), *Evidence based coaching handbook: Putting best practices to work for your clients* (pp. 219-253). Hoboken, NJ, US: John Wiley & Sons, Inc. |
| C. Kauffman, S. Joseph and A. Scoular | Leadership coaching and positive psychology | Positive Psychology in Practice: Promoting Human Flourishing in Work, Health, Education, and Everyday Life | To describe the theoretical orientations of coaching, examine their relations with applications of positive psychology, and outline potential contents of a positive psychology model of coaching | Organisational Contexts (Leadership) | Kauffman, C., Joseph, S., & Scoular, A. (2015). Leadership coaching and positive psychology. *Positive Psychology in Practice: Promoting Human Flourishing in Work, Health, Education, and Everyday Life*, 377-390. |
| C. Kauffman and A. Scouler | Toward a positive psychology of executive coaching | Positive psychology in practice | To describe the theoretical orientations of coaching, examine their relations with applications of positive psychology, and outline potential contents of a positive psychology model of coaching | Organisational Contexts (Executives) | Kauffman, C., & Scouler, A. (2004) Toward a positive psychology of executive coaching. In A. Linley & S. Josephs (Eds.), *Positive psychology in practice* (pp. 287–302)*.* Hoboken, NJ: John Wiley & Sons, Inc. |
| P.A. Linley and C. Kauffman | Positive coaching psychology: Integrating the science of positive psychology with the practice of coaching psychology | International Coaching Psychology Review | To provide some perspectives from empirical and theoretical work at the intersection of positive psychology and coaching psychology | General Life | Linley, P. A., & Kauffman, C. (2007). Positive coaching psychology: Integrating the science of positive psychology with the practice of coaching psychology. *International Coaching Psychology Review, 2*(1), 5-8. |
| P. A. Linley, L. Woolston and R. Biswas-Diener | Strengths coaching with leaders | International Coaching Psychology Review | To show how positive psychology, strengths approaches, and coaching psychology contribute to leadership strengths coaching programmes and practices | Organisational Contexts (Leadership) | Linley, P. A., Woolston, L., & Biswas-Diener, R. (2009). Strengths coaching with leaders. *International Coaching Psychology Review, 4*(1), 37-48. |
| D. N. Noble, K. Perkins and M. Fatout | On being a strength coach: Child welfare and the strengths model | Child and Adolescent Social Work Journal | To give examples of the strength coach approach in the context of child welfare | Social Work (Child-welfare) | Noble, D. N., Perkins, K., & Fatout, M. (2000). On being a strength coach: Child welfare and the strengths model. *Child and Adolescent Social Work Journal, 17*(2), 141-153. |
| L. G. Oades, T. P. Crowe and M. Nguyen | Leadership coaching transforming mental health systems from the inside out: The Collaborative Recovery Model as person-centred strengths based coaching psychology | International Coaching Psychology Review | To present the Collaborative Recovery Model (CRM) as a person-centred strengths based coaching framework | Organisational Contexts (Leadership) | Oades, L. G., Crowe, T. P., & Nguyen, M. (2009). Leadership coaching transforming mental health systems from the inside out: The Collaborative Recovery Model as person-centred strengths based coaching psychology. *International Coaching Psychology Review, 4*(1), 25-36. |
| J. Passmore and L. G. Oades | Positive psychology techniques: positive case conceptualisation | The Coaching Psychologist | To discuss the skill of positive case conceptualisation as a positive psychological technique | General Life | Passmore, J., & Oades, L. G. (2015). Positive psychology techniques: positive case conceptualisation. *The Coaching Psychologist, 11*(1), 43-45. |
| J. Passmore and L.G. Oades, L.G. | Positive psychology coaching: a model for coaching practice | The Coaching Psychologist | To discuss positive psychology coaching (PPC) as an approach suitable for use with coaching clients | General Life | Passmore, J., & Oades, L. G. (2014). Positive psychology coaching: a model for coaching practice. *The Coaching Psychologist, 10*(2), 68-70. |
| C.M. Sims | Second wave positive psychology coaching difficult emotions: Introducing the mnemonic of' ‘TEARS HOPE’ | The Coaching Psychologist | To illustrate the importance to maintain balance between the dark sides (weaknesses) along with the bright side (strengths) and to integrate the challenging and difficult aspects of human experience into our understanding of wellbeing and flourishing | General Life | Sims, C. M. (2017). Second wave positive psychology coaching difficult emotions: Introducing the mnemonic of' ‘TEARS HOPE’. *The Coaching Psychologist, 13*(2), 66-79. |
| F.W. Stander | Strength Coaching as an Enabler of Positive Athlete Outcomes in a Multi-cultural Sport Environment | Coaching Psychology: Meta-theoretical perspectives and applications in multicultural contexts | To introduce a practical strength-based coaching model that can be applied in the context of sport | Organisational Contexts (General) | Stander, F. W. (2016). Strength Coaching as an Enabler of Positive Athlete Outcomes in a Multi-cultural Sport Environment. In L. E. van Zyl, M. W. Stander, & A. Odendaal (Eds.). *Coaching Psychology: Meta-theoretical perspectives and applications in multicultural contexts* (pp. 279-298): Cham: Springer. |
| M. Tarragona | Positive psychology and life coaching | Positive Psychology in Practice: Promoting Human Flourishing in Work, Health, Education, and Everyday Life | To define life coaching and what sets it apart from executive coaching and psychotherapy | General Life | Tarragona, M. (2015). Positive psychology and life coaching. In S. Joseph (Ed.), *Positive Psychology in Practice* (pp. 249-264). Hoboken, NJ: John Wiley & Sons. |
| L. E. Van Zyl and M.W. Stander | A strengths-based approach towards coaching in a multicultural environment | Interdisciplinary handbook of the person-centered approach | To develop a strengths-based coaching model conducive to a multicultural environment | Organisational Contexts (General) | Van Zyl, L. E., & Stander, M. W. (2013). A strengths-based approach towards coaching in a multicultural environment. In J. H. D. Cornelius-White, R. Motschnig-Pitrik, & M. Lux (Eds.), *Interdisciplinary handbook of the person-centered approach* (pp. 245-257). New York, NY: Springer. |
| L. E. van Zyl, R. Motschnig-Pitrik and M. W. Stander | Exploring positive psychology and person-centred psychology in multi-cultural coaching | Coaching psychology: Meta-theoretical perspectives and applications in multicultural contexts | To contrast eclectic and purist theorising through examining similarities and differences between positive psychology and the person-centred approach as dominant paradigm perspectives within multi-cultural coaching | Organisational Contexts (General) | Van Zyl, L. E., Motschnig-Pitrik, R., & Stander, M. W. (2016). Exploring positive psychology and person-centred psychology in multi-cultural coaching. In L. E. van Zyl, M. W. Stander, & A. Odendaal (Eds.). *Coaching psychology: Meta-theoretical perspectives and applications in multicultural contexts* (pp. 315-355). Cham: Springer. |
| M. K. White and P. Barnett | A five-step model of appreciative coaching: a positive process for remediation | Remediation in Medical Education | To share experience of remediation coaches, describe the psychological foundations of appreciative inquiry and discuss the coaching context, roles, and process | Organisational Contexts (General) | White, M. K., & Barnett, P. (2014). A five step model of appreciative coaching: a positive process for remediation. In Kalet, A., & Chou, C. L. (Eds.) *Remediation in Medical Education* (pp. 265-281). New York, NY: Springer. |
| J. M. Yeager and K. H. Britton | Positive psychology coaching for sports leaders | Positive Psychology in Sport and Physical Activity | To demonstrate how Executive coaches with practices grounded in positive psychology can help sports leaders establish organisational cultures that contribute to higher performance through greater wellbeing | Professional Sports | Yeager, J. M., & Britton, K. H. (2017). Positive psychology coaching for sports leaders. In A. Brady & B. Grenville-Cleave (Eds.). *Positive Psychology in Sport and Physical Activity* (pp. 243-255): London, UK: Routledge. |
